# Exploiting ancestral mammalian genomes for the prediction of human transcription factor binding sites

**DOI:** 10.1186/1471-2105-13-S19-S2

**Published:** 2012-12-19

**Authors:** Mathieu Blanchette

**Affiliations:** 1 McGill Centre for Bioinformatics and School of Computer Science, McGill University, H3C 2B4, Québec, Canada; 2 This work was carried out in part while on sabbatical at the Laboratoire d'Informatique, Robotique, et Microélectronique de Montpellier (Université Montpellier 2), 161 rue Ada, Montpellier, France

## Abstract

**Background:**

The computational prediction of Transcription Factor Binding Sites (TFBS) remains a challenge due to their short length and low information content. Comparative genomics approaches that simultaneously consider several related species and favor sites that have been conserved throughout evolution improve the accuracy (specificity) of the predictions but are limited due to a phenomenon called binding site turnover, where sequence evolution causes one TFBS to replace another in the same region. In parallel to this development, an increasing number of mammalian genomes are now sequenced and it is becoming possible to infer, to a surprisingly high degree of accuracy, ancestral mammalian sequences.

**Results:**

We propose a TFBS prediction approach that makes use of the availability of inferred ancestral mammalian genomes to improve its accuracy. This method aims to identify binding loci, which are regions of a few hundred base pairs that have preserved their potential to bind a given transcription factor over evolutionary time. After proposing a neutral evolutionary model of predicted TFBS counts in a DNA region of a given length, we use it to identify regions that have preserved the number of predicted TFBS they contain to an unexpected degree given their divergence. The approach is applied to human chromosome 1 and shows significant gains in accuracy as compared to both existing single-species and multi-species TFBS prediction approaches, in particular for transcription factors that are subject to high turnover rates.

**Availability:**

The source code and predictions made by the program are available at http://www.cs.mcgill.ca/~blanchem/bindingLoci.

## Introduction

With the rapid increase in the number of fully or partially sequenced genomes [1] comes a wealth of evolutionary information that begs to be analyzed and investigated. The complete genomes of several groups of relatively closely related species (such as mammals [2], fruit flies [3], or yeasts [4]) have recently been compared to produce a mine of evolutionary information that has then been used to improve the accuracy of predictions of various types of functional elements of extant genomes such as protein-coding genes, RNA genes, transcriptional regulatory elements, and many others (reviewed in [5]). In this paper, we take advantage of another recent advance of comparative genomics, i.e. the high accuracy inference of nearly complete ancestral mammalian DNA sequences, to design a new approach for the identification of Transcription Factor Binding Sites (TFBS) in the human genome.

### Ancestral genome inference

An interesting prospect linked to the availability of a large number of extant genomes is the possibility of computationally inferring ancestral genomes. Assuming the availability of a set of extant genomes related to each other via a known phylogenetic tree, work has been done in that direction at different levels of resolution. First, the community has used high-level genome representations based on gene arrangements and focussed on the inference of ancestral gene orders and the history of rearrangements that has led to a given set of extant genomes (see for example [6-8], reviewed in [9,10]). Challenges at this level involve the computational complexity of most problem formulations, the difficulty to properly account for and weigh all types of evolutionary events possible, and the problem of properly identifying orthologs.

At a finer scale, which is the one we consider in this paper, studies have focussed on DNA sequence evolution at the level of substitutions, insertions, and deletions, and have asked to infer a set of ancestral sequences that are most likely (or most parsimonious) given a set of extant, collinear (non-rearranged) sequences. This inference problem is also challenging due to the difficulty of correctly aligning these sometimes highly divergent sequences, the computational complexity of maximum likelihood indel inference, etc. However, the significant amount of work invested in whole-genome multiple sequence alignment [11-13] and the set of exact and heuristic algorithms recently developed to infer ancestral DNA sequences from these alignments [14-18] now allow the inference of large sections of syntenic regions with good accuracy.

The eutherian mammals phylum is a particularly interesting target for ancestral genome inference, for several reasons. First, it includes our own human genome, so the study of ancestral genomes may shed some light on the function of various parts of our own genome. Second, due to the rapid radiation of a large number of eutherian lineages in a relatively short amount of time (the mammalian radiation) [19], certain early-eutherian mammal genomes can be inferred to a surprisingly high degree of accuracy. For example, Blanchette *et al. *showed using simulated sequence evolution that most of the euchromatic genome of the Boreoeutherian ancestor (the ancestor of all eutherian mammals except Afrotherians (e.g. elephants) and Xenarthans (e.g. sloths and armadillos)) can be inferred with 98-99% base-by-base accuracy from extant genome from each of the main lineages, most of which have now been sequenced [20]. Improved algorithms have since then been proposed [14-16] and may yield even higher accuracy.

### Identification of transcription factor binding sites

Transcription Factors (TFs) are proteins that bind specific short pieces of DNA (typically 6-15bp) and contribute to regulating the expression of one or more nearby genes. When sufficiently many binding sites for a given TF are known, the TF's sequence affinity can be represented using a position weight matrix (PWM) or more complex models [21], which can then be used to scan a given sequence in order to identify new candidate sites. However, the computational prediction of the set of binding sites for a given transcription factor remains very challenging, in large part because the affinity to the DNA binding site is only one of the many factors influencing the binding of a TF, while others, such as chromatin conformation [22], nucleosome positioning [23], and the presence/absence of co-factors [24], are much more difficult to predict and integrate. The consequence of this lack of information is a very low prediction specificity, with generally 95-99% of sites matching a PWM being false positives (i.e. being never bound by the TF) [21].

Recent high-throughput technologies such as ChIP-Chip [25] and ChIP-Seq [26] have eased the identification of genomic regions bound by a given TF. These technologies have allowed the genome-wide mapping of the binding sites of a good number of TFs in various species and cell types [27]. However, despite their rapidly decreasing cost, they remain only part of the solution: as TFBS vary significantly from cell type to cell type and depending on the conditions (due to changes in chromatin state and co-factors), the number of experiments that would be required to produce a comprehensive map of all existing binding sites for a given TF would be unmanageable. In addition, just like any other approaches, false-positives and false-negatives remain an issue, especially in highly repetitive regions of the genome [28]. In addition, the binding of a TF to a given site does not mean that this event has any consequences on gene expression. There thus remains a need for an improved computational TFBS predictor that would be cell-type independent and would focus on sites that actually affect gene expression, therefore complementing experimental data.

Phylogenetic footprinting is an important family of approaches aiming to improve the accuracy of computational TFBS prediction by making use of comparative genomics approaches [29-31]. The principle is simple: functional sites (i.e. true positive PWM hits) should tend to be more conserved across species than non-functional sites (i.e. false positive PWM hits), thanks to natural selection. Predictions based on the PWM scan of a single genome (e.g. the human genome) could in principle be improved by comparing a given region to its orthologs in other species, identifying regions whose mutation rates have been lower than expected for neutrally evolving sites, and focussing on PWM matches located in those regions. This type of approaches has indeed shown very good success [31], but accuracy has been limited by an evolutionary process called binding site turnover [32,33]. Because TFBS are very short stretches of DNA and because many TFs are fairly permissive in terms of the DNA sequence they bind to, a small number of mutations (substitutions or indels) can easily turn an unbound sequence into one that can be bound by a given TF. Because the function of TFBSs is often not highly dependent on their exact position w.r.t the regulated gene (within, say, a hundred base pairs), a newly created site that would be sufficiently close to an existing functional one may be able to regulate the target gene just as effectively as the original site.

The resulting pair of TFBS is thus redundant, so that a mutation hitting one or the other of the binding sites and destroying its ability to bind the TF would not be strongly deleterious, due to the presence of the other copy. The end result, though, is a sequence where neither site seems well conserved when compared to its orthologous regions in other species. The consequence is that, although function (in the form of the ability of the broad locus to bind the TF) might have been preserved throughout evolutionary time, the mechanisms by which it is implemented may have changed. The shorter and weaker the TFBS signal, the higher the turnover rate. Phylogenetic footprinting approaches not accounting for this phenomenon would thus be at risk of missing such sites and although a few approaches have indeed been proposed to deal with this problem [30], they have not been widely adopted.

### Paper outline

In this paper, we describe an approach that makes use of our ability to accurately infer ancestral genomic sequences in order to identify binding loci, i.e. regions of a few hundred base pairs that have preserved the ability to be bound by a given TF, while allowing for turnover within the region. We start by describing briefly our approach to ancestral genome inference. Then, we present a simple model of neutral sequence evolution where the number of predicted transcription binding sites in a given region changes over time due to sequence drift. Based on this model, we present an approach designed to assess the statistical significance of the level of TFBS content conservation of a given putative binding locus. Our approach is evaluated on a set of experimentally determined binding sites identified using ChIP-Seq (from the ENCODE project), and we show that it yields significant gains in prediction accuracy as compared to competing approaches, especially in the case of TFs that are subject to high rates of binding site turnover.

## Methods

### Ancestral genomic sequence inference

Ancestral mammalian genomic sequences were inferred as follows. A whole-genome multiple sequence alignment was first obtained from the UCSC genome browser [34]. This alignment, which includes the complete genomes of 33 mammals, was built using the blastZ/Multiz pipeline [35,36]. It is divided into a large number of syntenic blocks within which no rearrangements, duplications, or large insertions are expected to have happened. For each alignment block, ancestral sequences (for the phylogenetic tree of mammals used in [34]) were inferred at each internal node using the Ancestors 1.1 program [16], which infers the maximum likelihood ancestral sequences based on an evolutionary model including context-dependent substitutions, as well as insertions and deletions.

Because of the computational effort required to infer ancestral sequences, we limited our analysis to human chromosome 1 (with its corresponding fragments in other mammals), which consists of approximately 250 Mb of sequence in each species and ancestor, and constitutes roughly 8% of the human genome. Scaling up to the whole genome offers no particular challenge other than running time.

### Transcription factor binding site predictions

We used a set of 898 transcription factor PWMs available from either Transfac (version 9.2) [37] or Jaspar [38] to predict putative TFBS in each of the extant and predicted ancestral sequences. Scoring was performed using a simple log-likelihood ratio (LLR) approach [21]. For each TF, we tried to select a LLR score threshold so that most genuine sites are predicted as positive. To this end, for each TF, we randomly generated a set of 1000 sequences based on the probabilities defined by the PWM, calculated the LLR score for each sequence, and chose a LLR threshold that corresponds to the 10th percentile of the LLR scores (i.e. 900 of the sequences have a LLR score that exceed the threshold). The number of predicted binding sites for each TF varies from roughly 60 per kb for PWMs with low information content such as C/EBP (M00770) to 0.04 per kb for PWMs with high information content such as ELK4, with a median close to 2 per kb (obviously, the number of sites predicted for a given TF can be reduced by using a stricter score threshold). As previously mentioned, the vast majority of these predictions are expected to be false-positives, and the goal of the method proposed in this paper is to identify those that are the most likely to be functional.

### A neutral model of TFBS content evolution

We start by presenting a simple evolutionary model that describes how the *number *of predicted TFBS in a given *non-functional, neutrally evolving *genomic DNA sequence evolves over time. Consider a neutral evolution model M  that defines the probability of sequence *s*(*t*) to evolve into sequence *s*(*t *+ *δ*) after time *δ*. An ideal sequence evolution model should include not only substitutions, but also insertions, deletions, and other higher-level mutations such as duplications and genome rearrangements.

Now, consider a (deterministic) TFBS prediction algorithm A  that takes as input a DNA sequence s and a TF *T*, and identifies putative binding sites for *T *in s. For example, A  may scan s (on both strands) with a PWM for *T *and report every site whose log-likelihood score exceeds an appropriately chosen threshold. Let $B_{A}$ (*s*, *T*) be the number of sites predicted by A  in sequence *s*.

We are interested in the following question: if algorithm A  predicts a binding sites for TF *T *in sequence *s*(*t*), what is the probability that sequence *s*(*t *+ *δ*) will contain b predicted binding sites, i.e. $\text{Pr}\left[B_{A}\left(s\left(t+\delta \right),T\right)=\text{b}|B_{A}\left(s\left(t\right),T\right)\right)=a]$? Note that we only consider here the number of predicted binding sites for *T*, not their location or orientation. Our concern is the rate at which new binding sites are created and existing binding sites are lost, although we will not model each separately, but only their combined effects on the TFBS count. We emphasize that we are concerned with the change in predicted TFBS count in a random sequence neutrally evolving into another sequence. All TFBS predictions considered are therefore false-positives, and thus we model false-positive TFBS counts in neutrally evolving sequences. Deviations from this null model will suggest functionality.

Given a fully specified evolutionary model *M*, the desired probability $\text{Pr}\left[B_{A}\left(s\left(t+\delta \right),T\right)=\text{b}|B_{A}\left(s\left(t\right),T\right)\right)=a]$ could in principle be obtained by summing over the infinitely many ancestral sequences *s*(*t*) and evolutionary scenarios leading to the appropriate number of predicted sites. Instead of analytically calculating this complex expression (whose complexity depends on that of the evolutionary model M  and the binding site predictor A ), we estimate these probabilities empirically from actual sequence alignment data. Specifically, for each TF *T*, we consider a set of 1.2 million non-overlapping 200-bp windows taken from the non-coding portion of human chromosome 1, together with their orthologous and inferred ancestral sequences from placental mammals. Let us denote by *G_u_*(*W*) the portion of the genome at node *u *of the phylogenetic tree that is orthologous to region *W *of the human genome. For each window position *W *and each pair of adjacent nodes (*p*(*u*), *u*) in the phylogenetic tree (with *p*(*u*) being the parent of *u*), we first estimate the rate of sequence divergence between *G*_*p*(*u*)_(*W*) and *G_u_*(*W*) based on percent identity of the two aligned sequences, from which divergence estimate λ(*G*_*p*(*u*)_(*W*), *G_u_*(*W*)) is obtained. We then apply TFBS prediction algorithm A  to both *G*_*p*(*u*)_(*W*) and *G_u_*(*W*), to obtain *B*(*G*_*p*(*u*)_(*W*), *T *) and *B*(*G_u_*(*W*), *T*). Finally, the desired probability is empirically estimated as

$$\begin{array}{c} Pr\left[B_{A}\left(s\left(t+\delta \right),T\right)=b|B_{A}\left(s\left(t\right),T\right)=a\right]=\\ \;\;\;\;\;\frac{\left|\left\{W,u|\lambda \left(G_{p\left(u\right)}\left(W\right),G_{u}\left(W\right)\right)=\lambda ,B\left(G_{p\left(u\right)}\left(W\right),T\right)=a,B\left(G_{u}\left(W\right),T\right)=b\right\}\right|}{\left|\left\{W,u|\lambda \left(G_{p\left(u\right)}\left(W\right),G_{u}\left(W\right)\right)=\lambda ,B\left(G_{p\left(u\right)}\left(W\right)\right)=a\right\},)\right|}, \end{array}$$

where all possible non-coding windows *W *and tree branches are considered. Ne note that for practical reasons, divergence estimates are rounded to the nearest percentage point before testing for equality. A small fraction of these windows (probably less than 1%) will actually contain functional binding sites for *T *and will slightly taint our neutral model. However, this fraction is sufficiently small that its impact is very minor. Furthermore, the bias caused by the presence of functional sites will only result in our final predictions being slightly over-conservative.

In addition to its relative simplicity, this empirical estimation has a number of advantages. First, it does not require the explicit specification of an evolutionary model, although one is required for the inference of ancestral genomes. Second, as it is based on the actual multiple sequence alignment used later on for TFBS prediction, it naturally includes variation due to misalignments and incorrect ancestral sequence prediction.

### Binding loci prediction

Equipped with a neutral model of predicted TFBS content, we can now consider a particular region of the human genome (on chromosome 1), together with its aligned orthologous and ancestral sequences, and ask whether, in that region, the TFBS count for a given TF *T *seems to evolve under the neutral model, which would suggest that the predicted sites are false-positives, or whether there is evidence for selection to preserve the number of binding sites over time, which would be an indication that those predicted sites may be functional. We considered and tested several alternatives for the precise form of this hypothesis test before settling on the following. A key question was whether (1) this analysis should be conditioned on the observed degree of sequence similarity of the region under consideration along a particular branch, or whether (2) the level of sequence divergence associated with a given branch of the tree should be considered fixed. In the latter case (2), a region with an extremely low sequence divergence (e.g. a sequence that would be 100% conserved in all orthologous and ancestral sequences) would exhibit perfect conservation of TFBS content for every predicted sites it contains. This may be appropriate if the sequence conservation is indeed due to selective pressure to maintain these sites. However, if the region is conserved for other reasons (e.g. it is an unannotated non-coding RNA), this would result in a large number of TFBS false-positive predictions. In the former case (1), the observed sequence conservation between pairs of sequences is considered as given, and the surprise associated with the degree of conservation of predicted TFBS is conditioned on it. Sequences with perfect sequence conservation can thus result in no surprising TFBS conservation. Only somewhat divergent sequences can yield predictions. This is in some sense similar to the approach taken for RNA secondary structure prediction based on comparative genomics [39], where it is the presence of compensatory mutations that results in predictive power. We elected to use approach (1) as it is likely to result in fewer false positive predictions. The drawbacks are discussed in Results.

**Figure 1:**
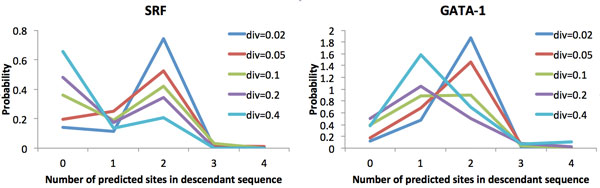
**Examples of the distribution of the number of predicted TFBS in the descendant of a 200-bp ancestral sequence containing two predicted sites that diverge for various durations, for a TF with a relatively high information content matrix (SRF; left) and one with a relatively low information content (GATA-1; right)**. At low divergence values, the descendant sequence still contains two sites with high probability. As the level of divergence increases, the distribution of the number of predicted sites converges toward a different stationary distribution for each TF.

Given a region *W *of the human genome and orthologs and ancestral sequences, we calculate a score based on the p-values of the level of predicted TFBS count conservation along each branch of the tree. For every tree node *u *(other than the root) with parent *p*(*u*), we obtain the conditional p-value

$$\begin{array}{c} pvalue\left(W,T,u\right)=\\ \;\;\;\;Pr[B_{A}\left(s\left(t+\delta \right),T\right)-B_{A}\left(s\left(t\right),T\right)\geq B_{A}\left(G_{p\left(u\right)}\left(W\right),T\right)-B_{A}\left(G_{u}\left(W\right),T\right)|\\ \;\;\;\;\;\;\;\;\;\;\;\;B_{A}\left(s\left(t\right),T\right)=B_{A}\left(G_{p\left(u\right)}\left(W\right),T\right),\delta =\lambda \left(G_{p\left(u\right)}\left(W\right),G_{u},\left(G\right)\right)], \end{array}$$

which measures the probability that a random sequence containing *B*(*G*_*p*(*u*)_(*W*), *T*) predicted binding sites and evolving neutrally for *δ *= *λ*(*G*_*p*(*u*)_(*W*), *G_u_*(*W*)) time will have preserved its number of binding sites to at least the same degree as has been observed in the actual pair of sequences. Tree branches with small p-values correspond to branches where the observed variation in TFBS counts is lower than expected for neutrally evolving sequences, suggesting that natural selection may be at work preserving the predicted sites. However, as shown in Figure 1, p-values obtained for individual tree branches are rarely sufficiently small to reliably suggest deviations from the neutral evolution model. Instead, we combine the p-values obtained over the branches of the whole tree to obtain a global binding locus score for the region W and TF T under consideration:

$$Binding\;Locus\;Score\left(W,T\right)=-\underset{u\in tree}{\sum }\text{log}\left(pvalue\left(W,T,u\right)\right).$$

Finally, for each TF, the human genome (together with its aligned orthologs and ancestors) is scanned using a sliding window of size 200 (with 20bp offsets) and binding locus scores are recorded. The size of the window was selected to optimize overall agreement with ChIP-Seq experimental data, although it is possible that different TFs would be best predicted using different window sizes. A given window *W *is deemed interesting for TF *T *if its binding locus score is unlikely to have arisen by chance under the null model, which is evaluated by computing the convolution of the (discrete) p-value distributions (under the null) from each branch.

## Results

### Gains and losses of predicted TFBS in evolving sequences

We first assess the tendency for the number of predicted binding sites for a given TF to diverge as a function of evolutionary time. As sequences diverge more and more, the distribution of the number of predicted TFBS in the neutrally evolving descendant sequence tends toward its stationary distribution, which, for most TFs, means that with fairly high probability the window will contain no site at all. This tendency to lose binding sites is accelerated for TFs with long, highly specific PWMs, which are easily destroyed by point mutations or indels but rarely created by them. Figure 1 (left) illustrates this phenomenon for the SRF TF (Transfac M01007), which has a 9-bp, information rich PWM (information content = 15.1 bits), for the example where the parent sequence contains two binding sites. Loss of binding sites is slower and TFBS count typically do not reach as low levels for TFs with more degenerate PWMs, as new matches to those PWMs are more likely to arise through random mutations. This is illustrated in Figure 1 for GATA-1 (Transfac M00347), a TF with a relatively weak PWM (information content = 12.1 bits).

### Accuracy of binding loci prediction

Transcription factor binding loci were predicted in human chromosome 1, for each of 898 transcription factors for which a PWM was available in Transfac [37] or Jaspar [38], as described in Methods. For each TF, genomic regions of 200-bp were ranked based on their binding locus score. For a given threshold of binding locus score, our algorithm predicts as functional TFBS all PWM hits located within loci of sufficient score. Additional file 1 shows an example of one of the strongest predicted binding loci for the GATA-1 TF, which is confirmed by ChIP-Seq data (see below), but which does not show a high degree of sequence conservation due to a significant amount of TFBS turnover. Binding loci for each TF are available at http://www.cs.mcgill.ca/~blanchem/bindingLoci.

We compared our TFBS prediction approach to two other simpler approaches. The first, that we call the single-genome PWM scanning, simply consist in scanning the human genome with a PWM and identifying sites whose log-likelihood score exceeds a given threshold. The second, called PWM scanning of conserved regions, posits that functional sites are more likely to be found within genomic regions that are exhibiting a high degree of sequence conservation between mammals. Siepel *et al. *have developed PhastCons [40], a tool that, from a given whole-genome multiple alignment, identifies genomic regions whose reduced substitution rate suggests that they may be under selection. The set of conserved regions identified by PhastCons on the basis of the alignment of eutherian mammal genomes used in this study consists of 335,083 regions of approximately 30bp on average, covering 4.7% of human chromosome 1. These regions have been shown to be strongly enriched for cis-regulatory elements [27,34,40]. The set of predictions made by the PWM scanning of conserved regions approach is the restriction of the single-genome PWM predictions to the set of conserved regions identified by PhastCons.

To evaluate the ability of our approach to identify functional TFBSs, we used a collection of binding loci identified by ChIP-Seq through the ENCODE project [27]. ChIP-Seq identifies regions bound by a given TF but lacks the resolution to pinpoint exactly each TFBS. Instead, it identifies regions of 100-300bp that are bound by a given TF in the sample under consideration. ChIP-Seq data were available for 35 TFs (in a handful of cell types) for which PWMs were available (data produced by the Richard Myers Lab, available from the UCSC Genome Browser [41]). Most TFs have between 500 and 5000 bound regions on chromosome 1, which cover between 0.07% and 0.7% of the chromosome. The set of 35 TFs was divided into two functionally different groups. The first (that we informally label non-developmental TFs) is a group of 20 TFs involved in basal regulation (e.g. SP1, YY1), cell-cycle regulation (e.g. E2F1), cell growth (e.g. GATA-1, c-Myc), or immune response (NFkB). The second (labeled as developmental TFs) is a set of 15 TFs that play key roles in regulating embryonic development and cell differentiation, or that regulate tissue-specific or hormone-dependent expression patterns.

**Figure 2:**
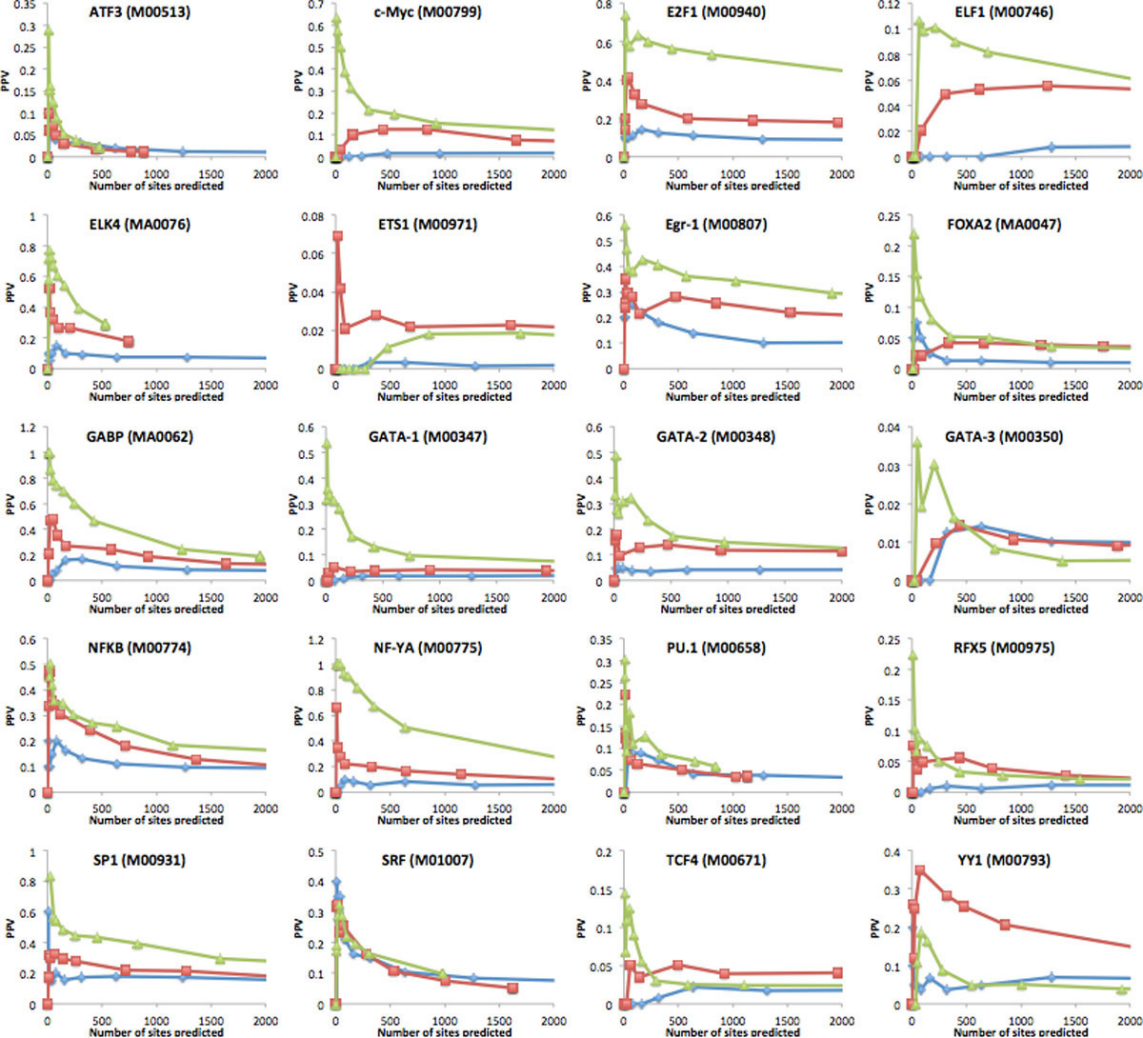
**Positive predictive value (fraction of bases within predicted sites that overlap a ChIP-Seq peak for the same TF) curves for 20 non-developmental TFs, as a function of the number of TFBS being predicted**. Three prediction approaches are considered: (i) Blue curves: PWM scanning; (ii) Red curves: PWM scanning limited to highly conserved regions identified by PhastCons on the eutherian genomes alignment; (iii) PWM scanning limited to regions identified as conserved binding loci by our algorithm. For the red and blue curves, the desired number of predicted sites is obtained by varying the LLR threshold (but always maintaining it above the minimum threshold chosen for each TF). For the green curves, the desired number of predicted sites is obtained by varying the threshold on the binding locus scores and reporting all sites with LLR score above the minimum threshold located within these regions.

**Figure 3:**
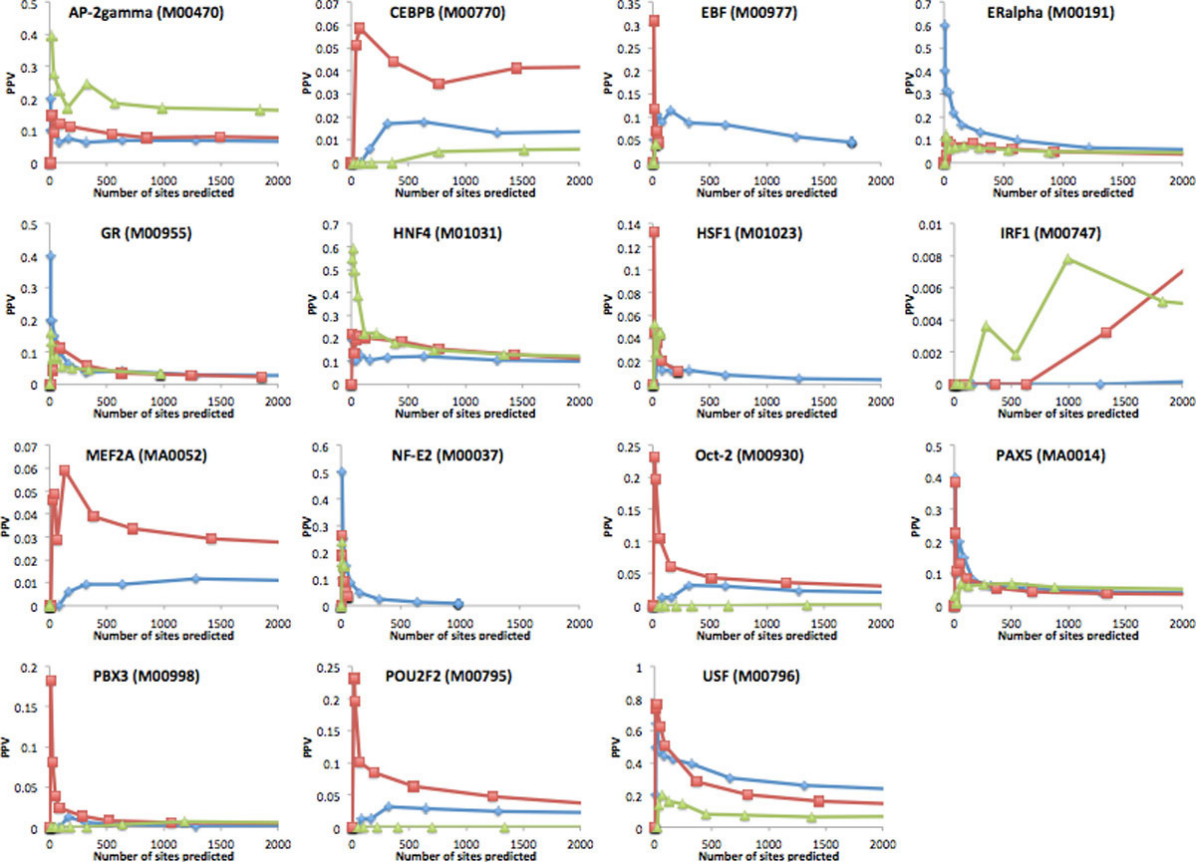
**Positive predictive value curves for 15 developmental/hormone response TFs, as a function of the number of TFBS being predicted**. See caption of Figure 2 for details. In some cases (e.g. NF-E2, MEF2A, EBF), very few binding loci were identified by our algorithm, resulting in the impossibility of that approach to make more than a handful of TFBS predictions, explaining why the green curve does not extend over the full range of the x axis.

Figures 2 (for non-developmental TFs) and Figure 3 (for developmental TFs) show the positive predictive value (PPV, defined as the fraction of TFBS predictions that overlap a region identified by ChIP-Seq) obtained by each of the three approaches, as a function of the number of sites predicted (i.e. for different prediction score thresholds). For 15 of the 20 non-developmental TFs considered, there is a clear gain of PPV obtained by considering only PWM hits located within our high scoring binding loci, as compared to the two other approaches. In many cases, the improvement in accuracy for high-confidence predictions (top 100 TFBS predicted) in the range of 2 to 4 fold. We note that simple PWM scanning without guidance by either PhastCons or binding loci (blue curves in Figure 2) is rarely competitive with the two other approaches, except in the rare cases (PU.1, SRF) where the information content of the PWMs is high enough to make predictions based on LLR scores alone fairly reliable. We observe that the accuracy of the predictions varies significantly from TF to TF. For some (NF-YA, SP1, E2F1, GABP), it is possible to predict more than 500 sites (on chromosome 1 alone) and retain an accuracy above 50%. For others (c-Myc, Gata-1/2), the top 100 predictions show the same level of reliability, but the accuracy drops quickly as less confident predictions are considered. Finally, certain TFs (RFX5, FOXA2, GATA-3) obtain very low accuracy predictions with all methods, suggesting that the PWM used may be of low quality. The results are quite different for TFs that are involved in regulating more complex processes such as embryonic development, tissue-specific expression, or hormone response (see Figure 3). These TFs often interact with each other to bind enhancers and form cis-regulatory modules [42,43]. These modules tend to be highly conserved across species due to their critical role [44,45]. For example, the overlap between PhastCons conserved regions and ChIP-Seq regions for developmental TFs POU2F2, MEF2A, OCT-2, and PBX3 is much higher that of ChIP-Seq regions for non-developmental TFs SP1, GATA-1 or NF-YA (15-18% vs 9-11%). In these cases, PWM scanning limited to highly conserved regions is more effective than our approach. This is in part due to the fact that highly conserved regions are indirectly penalized by our approach because they lack the sequence divergence required to reveal unexpected levels of TFBS count conservation (recall that our calculations are conditioned on the observed degree of sequence divergence). Indeed, the few non-developmental TFs for which our approach performs poorly (ETS1 and YY1) have ChIP-Seq determined binding sites that are also enriched in PhastCons conserved regions (overlap of 17.8% and 25.0% respectively). We note however that for the majority of developmental TFs, the PPV of all methods is quite low.

## Discussion and future work

Whereas selection operates on genomic DNA to preserve fitness, the precise mechanism through which a DNA sequence encodes a desirable phenotype (e.g. the activation of a gene by a TF) can evolve over time via binding site turnover, resulting in sites whose position-specific evolutionary rates, if measured over a large phylogenetic tree such as the mammalian phylogeny, may not differ substantially from the neutral rate. Although a large number of approaches exist for the detection of regulatory regions based on multi-species comparison, our approach is the first to make use of ancestral sequences to track the potential of a given region to bind a TF over evolutionary time. The regions identified as likely binding loci for a TF are often not the most conserved ones at the sequence level (e.g. for the GATA-1 and c-Myc TFs, only 18% and 40% and of top-scoring loci overlap PhastCons elements), but it is the level at which they have maintained the ability to bind the TF that makes them stand out. Our approach is thus complementary to the more classical phylogenetic footprinting approaches that seek binding site conservation as evidence for function. Indeed, our approach produces significantly improved prediction accuracy for a large number of TFs that tend to bind promoters that exhibit low to medium sequence conservation, while other approaches are preferable for developmental TFs whose binding sites lie within large, highly conserved enhancers. Clearly, a more detailed benchmarking of the panoply of approaches that have been proposed for the identification of TFBS based on comparative approaches would be timely, although it exceeds the scope of this paper.

Several directions are worth exploring from here. An interesting avenue would be to make use of the full posterior probability distribution over ancestral sequences, produced by Ancestors 1.1 for each ancestral node in the tree in the form of a sequence profile, in order to estimate more accurately the expected number of sites contained within a given ancestral region. Replacing the TFBS count in the maximum likelihood ancestral sequence by an expected TFBS count may be particularly beneficial when there is considerable uncertainty about a given ancestral sequence. Doing so would necessitate the calculation of the expected number of PWM hits in a sequence generated by a given profile hidden Markov model (HMM), which can be done either analytically by coupling the profile HMM and the PWM-HMM, or, more pragmatically, by sampling ancestral sequences from the profile HMM. Another exciting prospect is to simultaneously consider groups of interacting TFs that coordinately bind DNA, and study the conservation of the binding potential of the group rather than that of individual TFs.

Genomic regions such as protein-coding exons, whose GC-content differs from that of the rest of the genome and where selection operates to maintain this unusual composition, are likely to yield an elevated rate of false-positive predictions for TFs whose binding affinity matches the GC composition. Future versions of our approach will derive different TFBS gain/loss models for regions with different GC content. An important assumption underlying our methodology is that the binding preference (modelled by a PWM) of a given TF remains constant across all extant and ancestral species considered. The same assumption underlies most comparative genomics studies [3,46] and appears well justified when the species considered are relatively closely related (e.g. within mammals or within drosophila). However, our approach could be modified to take into account different PWMs for different species, should those become available, by using the appropriate species-specific PWM when available, or, otherwise, that of the most closely related species.

Finally, the type of approaches described in this paper, where conservation of function potential, rather than sequence conservation per se, is evaluated, may be fruitfully applied to the identification of other types of functional elements whose position in the genome tends to be approximately conserved but whose exact location may vary due to turnover-like phenomena. This may be the case for splicing regulatory elements [47], which have similar properties to TFBS, as well as for more diffuse signals such as nucleosome positioning [48], which is defined by low-level sequence features that affect DNA flexibility.

## Competing interests

The author declares that he has no competing interests.

## Supplementary Material

Additional file 1 **Example of the GATA-1 binding sites predicted in each extant and ancestral sequences in the first exon of the NPL gene**. A region bound by GATA-1, identified by ChIP-Seq, is located just upstream of the second (alternative) exon. Rows corresponding to ancestral sequences are identified by listing the names of the extant descendants of each ancestor. Panel B is a zoom on the high-scoring binding locus region. Note that the region does not exhibit elevated sequence conservation, and contains many examples of TFBS turnover. Click here for file
