# Supplementary figures and images for: Exploiting ancestral mammalian genomes for the prediction of human transcription factor binding sites
Source: BMC Bioinformatics. 2012 Dec 19;13(Suppl 19):S2. doi: 10.1186/1471-2105-13-S19-S2 (PMC3526440; doi:10.1186/1471-2105-13-S19-S2)

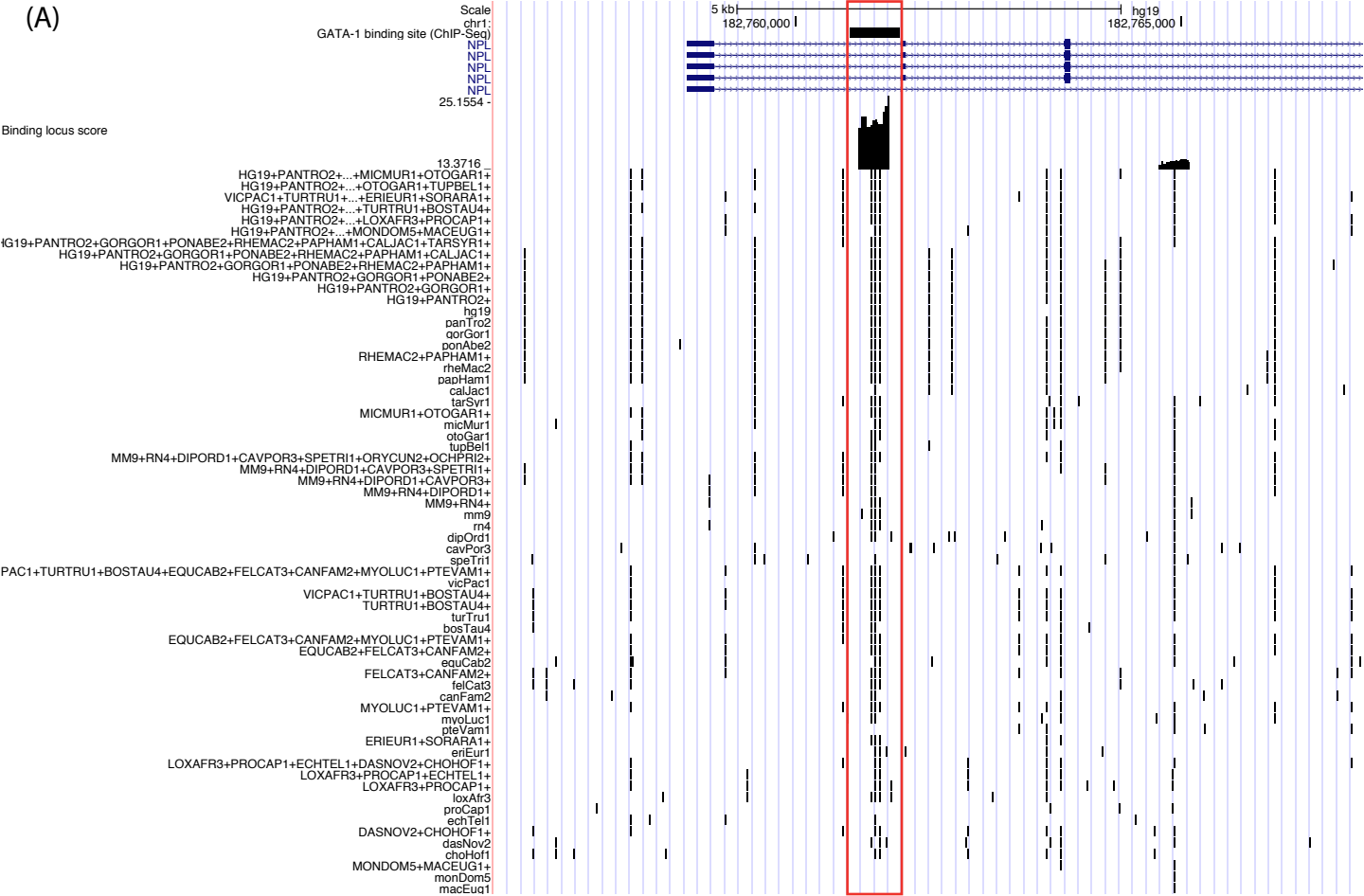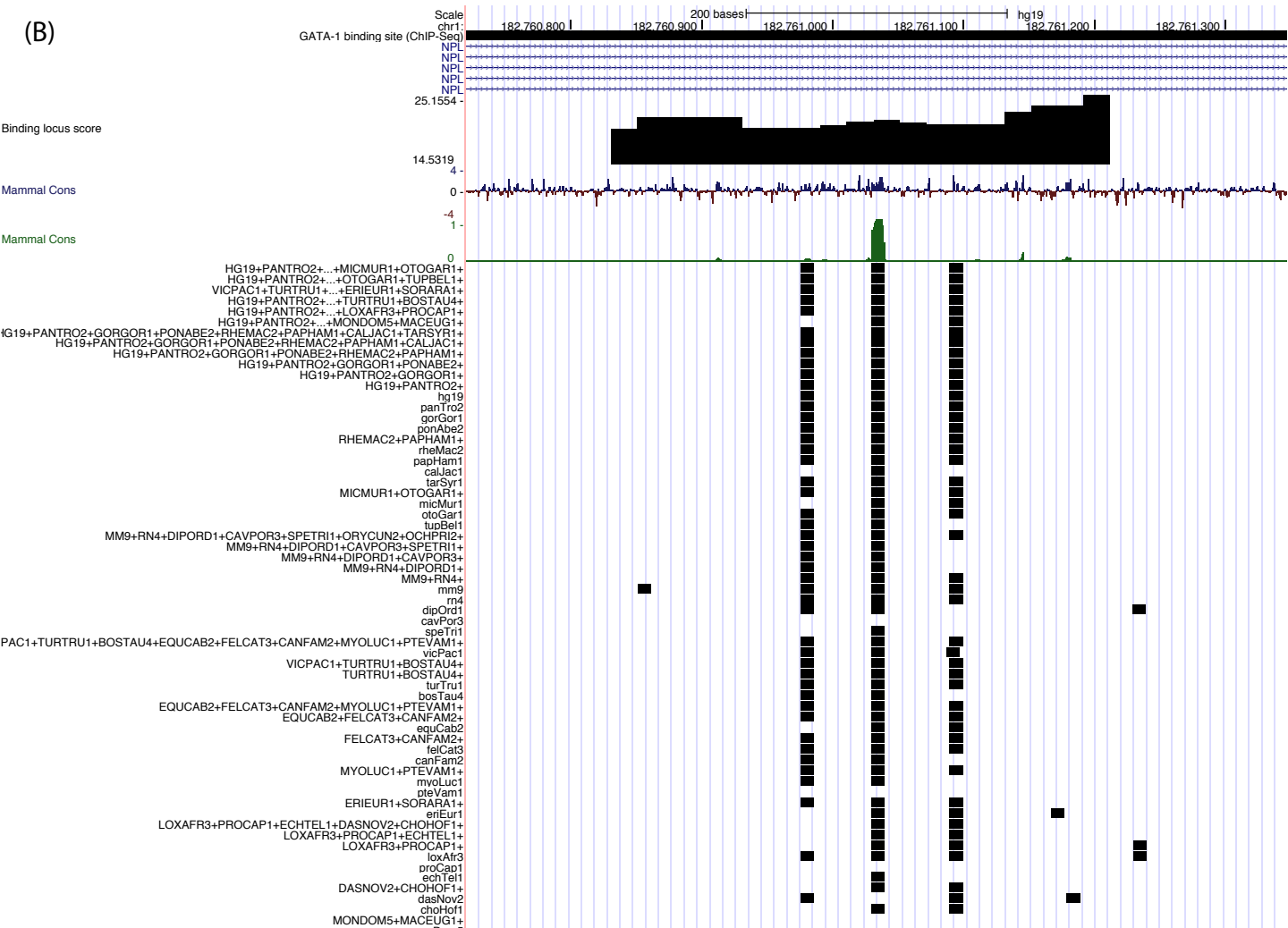

Supplement: Additional file 1 — Example of the GATA-1 binding sites predicted in each extant and ancestral sequences in the first exon of the NPL gene. A region bound by GATA-1, identified by ChIP-Seq, is located just upstream of the second (alternative) exon. Rows corresponding to ancestral sequences are identified by listing the names of the extant descendants of each ancestor. Panel B is a zoom on the high-scoring binding locus region. Note that the region does not exhibit elevated sequence conservation, and contains many examples of TFBS turnover. [file 1471-2105-13-S19-S2-S1.PDF]
